# Prevalence of common mental disorder and its association with perceived stigma and social support among people living with HIV/AIDS in Ethiopia: a systematic review and meta-analysis

**DOI:** 10.1186/s13033-024-00641-x

**Published:** 2024-07-08

**Authors:** Bereket Duko, Yitagesu Belayhun, Asres Bedaso

**Affiliations:** 1 School of Public Health, College of Medicine and Health Sciences, Hawassa University, Hawassa, Ethiopia; 2 Australian Centre for Precision Health, Unit of Clinical & Health Sciences, University of South Australia, Adelaide, SA 5000 Australia; 3 Curtin School of Population Health, Curtin University, Kent St, Bentley, Perth, WA 6102 Australia; 4 South Australian Health and Medical Research Institute, Adelaide, SA 5000 Australia; 5 Health Development Planning and Economic Administration, South Ethiopia Regional State Health Bureau, Jinka, Ethiopia; 6 The Daffodil Centre, University of Sydney, a joint venture with Cancer Council NSW, Sydney, Australia

**Keywords:** Common mental disorders, Psychological distress, Epidemiology, Prevalence, Associated factors, Determinants, Ethiopia

## Abstract

**Background:**

When common mental disorders (CMD) co-occur with HIV/AIDS, they can complicate patient diagnosis, help-seeking behaviors, quality of life, treatment outcomes, and drug adherence. Thus, estimating the pooled prevalence of CMD and its association with perceived stigma and social support among people living with HIV/AIDS (PLWHA) in Ethiopia could potentially support policymakers and health professionals to understand the disease burden and set a solution to improve the mental well-being of PLWHA.

**Methods:**

Popular databases such as PubMed, SCOPUS, EMBASE, and Psych-INFO as well as Google Scholar, AJOL, CINAHL, PILOTS and Web of Science were searched for the relevant articles conducted in Ethiopia. We included cross-sectional, case–control, and cohort studies in the review. The Comprehensive Meta-Analysis software version 3.0 was used to pool the results of the included studies. The Q- and I^2^-statistics were used to assess the heterogeneity between the included studies. We employed a random-effects meta-analysis model to estimate the pooled prevalence of CMD and to account for heterogeneity among the included studies. We also conducted a leave-one-out analyses, and stratified meta-analyses by gender (male and female).

**Results:**

The studies included in this systematic review and meta-analysis were published between 2009 and 2021, recruiting a total of 5625 participants. The pooled estimated prevalence of CMD among PLWHA in Ethiopia was 26.1% (95% CI 18.1–36.0). The pooled estimated prevalence of CMD was significantly higher among females, at 39.5% (95% CI 21.2–39.0), compared to males, 26.9% (95% CI 15.6–31.7). Moreover, the pooled estimated prevalence of CMD in PLWHA ranged from 23.5 to 28.9% in the leave-one-out sensitivity analysis, indicating that the removal of any single study did not significantly affect the pooled estimate. The pooled effects (AOR) of Perceived HIV stigma and poor perceived social support on common mental disorder were 2.91, 95% CI (1.35–6.29) and 5.56, 95% CI (1.89–16.39), respectively.

**Conclusion:**

People living with HIV/AIDS (PLWHA) who received poor social support and those with HIV-related perceived stigma were found to have strong association with CMD. Therefore, it is advisable that all PLWHA attending ART clinic should be screened for CMD, social support and HIV-related perceived stigma.

**Supplementary Information:**

The online version contains supplementary material available at 10.1186/s13033-024-00641-x.

## Background

Common mental disorder (CMD) refers to a group of mental health disorders that include depression, anxiety, and somatoform disorders with a significant contribution to the burden of disease in the middle- and low-income countries [1].  According to systematic review and meta-analysis of 174 surveys across 63 countries  in 2014, the global lifetime prevalence of CMD was 29.2% [2]. CMDupsurges the risk of emerging both communicable and non-communicable diseases in all age groups of the general population [3]. CMD is frequently reported among HIV infected individuals and it is the leading cause of infirmity among PLWHA [4–6].

Based on the reports from studies conducted in the low and middle-income countries (LMICs), the prevalence of CMD was found to be high [7–9]. For example, a study conducted in Zimbabwe using the Shona Symptom Questionnaire (SSQ14 >  = 9) reported 68.5% prevalence of CMD s among PLWHA (7). In contrast, another study from South Africa showed that 23.9% of people living with HIV reported symptoms of CMD [8]. Further, finding from the Nigerian study that used the Kessler Psychological Distress Scale (K10) to assess CMD reported 47.9% of PLHIV participants scored ≥ 20, suggesting CMD  [9].

Female gender, poverty, and stressful life events were found to be common determinants of CMD in non-HIV populations [10]. In other studies, correlates of CMD in PLWHA include the death of a significant other [11], family history of mental illness, poor coping style, alcohol dependency, food insecurity [12], exposure to negative life events [7, 13], posttraumatic stress disorder (PTSD) and perceived HIV stigma [14–16]. Additionally, factors such as poor social support, not disclosing HIV status, stressful feelings about the illness were significant provoking factors [17, 18].

Research has shown that individuals with CMD experience accelerated progression from HIV to AIDS [19]. Additionally, these individuals may have difficulty adhering to ART treatment [20], which can lead to increased viral load and death in patients with AIDS  [21, 22]. However, effective management of CMD has been found to improve the health and quality of life of PLWHA  [22, 23]. Although several studies have been conducted in Ethiopia to assess CMD among PLWHA [17, 24–33], there is significant inconsistency in the prevalence of CMD across the studies in the topic. Furthermore, there have been no previous systematic reviews or meta-analyses conducted on this topic in Ethiopia. Therefore, this review aimed to systematically review previous studies, summarize the magnitude of CMD, and examine their association with HIV-related perceived stigma and social support among PLWHA in Ethiopia. This review also aimed to formulate recommendations for future better clinical services.

## Methods

### Search strategy and selection process

We followed the Preferred Reporting Items for Systematic Reviews and Meta-Analyses (PRISMA) guidelines to conduct this systematic review and meta-analysis [34]. A predesigned study protocol for database searching, data extraction, inclusion–exclusion criteria, and quality evaluation was used. PubMed, SCOPUS, EMBASE, and Psych INFO databases were searched for relevant articles that assessed the prevalence of CMD among PLWHA in Ethiopia using the following search terms and keywords: (epidemiology OR prevalence OR magnitude) AND (common mental disorders OR psychological distress OR common mental illness OR psychiatric morbidity OR mental health problems) AND (associated factors OR correlates OR risk factors OR determinants) AND (people living with HIV/AIDS OR HIV patients OR HIV/AIDS) AND Ethiopia. Furthermore, we searched EMBASE, SCOPUS, and Psych INFO using database-specific subject headings. The search yielded relevant articles that were assessed for inclusion in the study. We also searched for articles indexed in Google Scholar, African Index Medicus, African Journals Online (AJOL), CINAHL, PILOTS and Web of Science.

### Eligibility criteria

We included cross-sectional, case–control, and cohort studies conducted either in community or institutional settings and assessed the prevalence and factors associated with CMD s or psychological distress among PLWHA  in Ethiopia. Commentaries, editorials, reviews, and letters to editors were excluded from the review.

### Methods for data extraction and quality assessment

Two independent reviewers (BD and AB) conducted data extraction based on the predefined data extraction form. The data extraction form included the authors' names, year of publication, sample size, study design, study setting, and the instrument used to measure common mental disorders as well as associated factors along with adjusted odds ratios. The Newcastle–Ottawa Scale (NOS), adapted for cross-sectional studies was used to check the methodological quality of studies included in the review [35]. This tool has been used in previous studies [36, 37]. The NOS scale assessed the quality of studies based on methods, sample size, sample representativeness, and comparability between participants. The agreement between the evaluators was appraised using the unweighted kappa statistic (YB and AB). The levels of agreement were categorized as poor (0), slight (0.01–0.20), fair (0.21–0.40), moderate (0.41–0.60), substantial (0.61–0.80), and almost perfect (0.81–1.00) [38].

### Data synthesis and analysis

We systematically reviewed qualitative data, including the identification of studies, study characteristics, and the quality of the included studies. Comprehensive Meta-Analysis software version 3.0 was used to conduct a meta-analysis, employing a random-effects meta-analysis model to pool the overall prevalence of CMD among PLWHA in Ethiopia [39]. We also computed pooled adjusted odds ratio (AOR) for factors associated with CMD  among PLWHA. The Q- and the I^2^-statistics were used to assess the heterogeneity between the studies [40], with values of 25, 50 and 75% indicating low, low, medium and high level of heterogeneity, respectively [40]. Publication bias was evaluated by using Egger’s test and visual inspection of funnel plot [41]. The level of significance was set at P < 0.05. Furthermore, we conducted a meta-regression to quantify the impact of the screening tools used to measure CMD, gender, and region a study originated on the observed heterogeneity across the studies included in the review.

## Results

### Identification of studies

**Fig. 1:**
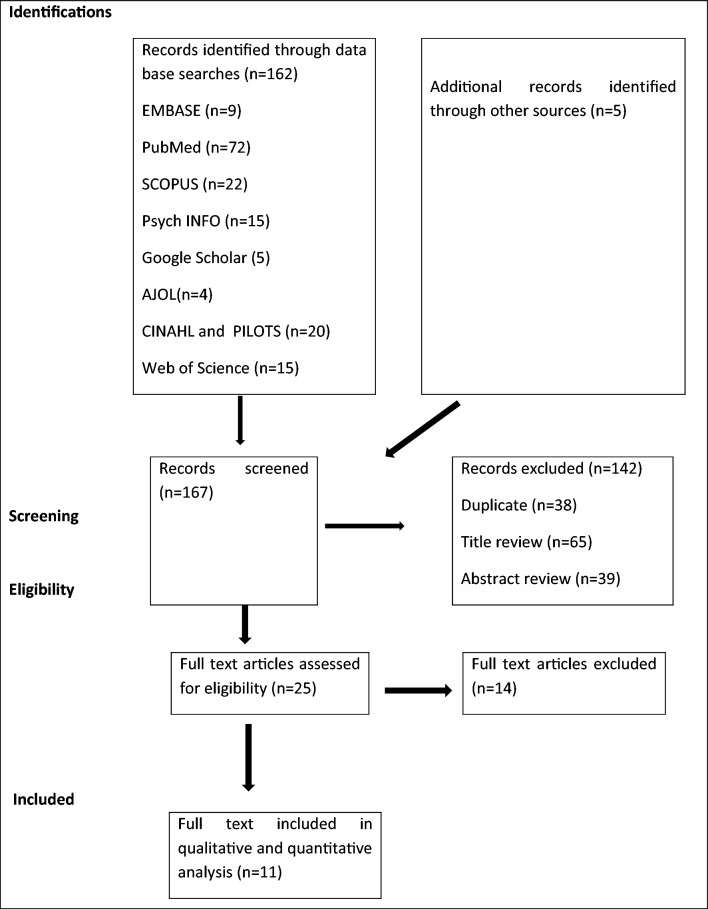
PRISMA flowchart of review search

A total of 162 articles were identified through electronic database searching. Besides, five more articles were obtained from references of the included articles. Out of the 167 articles, 142 were excluded as they did not meet the eligibility criteria (Fig. 1). Subsequently,  25 articles were selected for further screening, out of which 14 full text articles were excluded. Finally, 11 full-text articles were included in the final systematic review and meta-analysis.

### Characteristics of included studies

The studies included in the current systematic review and meta-analysis were published between 2009 and 2021 and recruited a total of 5625 study participants. Ten studies employed a cross-sectional study design, whereas one study used a cohort study design. Two studies were conducted in South Nation Nationalities and People Regional States, three in the Amhara region, five studies in the Oromia region, and one in the Harar region. Four studies used the Self-Reporting Questionnaire item 20 (SRQ20), one study used Hospital Anxiety and Depression scale (HADS) and six studies used the Kessler scale (K-10) (Table 1).

**Table 1:** Characteristics of studies included in the review

Study name	Sample size	Type of outcome	Study design	Region	Data collection tool	Prevalence in male (if any)	prevalence in female (if any)	Prevalence (%)	Quality score
Tesfaye and Bune [17]	500	Psychological distress	CS	SNNPR	HADS	NA	NA	11	8
Motumma A et al. [28]	420	Common mental disorder	CS	HARAR	SRQ20	24.80	29.20	28	8
Duko B et al. [26]	294	Common mental disorder	CS	SNNPR	SRQ20	44.30	30.20	32.7	9
Zewdu S et al. [25]	417	Common mental disorder	CS	Amhara	SRQ20	NA	NA	24.3	7
Basha EA et al. [24]	422	Psychological distress	CS	Amhara	SRQ20	3.80	10.20	7.8	7
Deribew A et al. [27]	465	Common mental disorder	CS	Oromia	Kessler scale-10	53.40	48.00	46.7	9
Deribew A et al. [29]	465	Common mental disorder	Cohort	Oromia	Kessler scale-10	NA	NA	18.6	9
Soboka M et al. [30]	389	Common mental disorder	CS	Oromia	Kessler scale-10	NA	NA	45.2	7
Soboka M et al. [31]	389	Common mental disorder	CS	Oromia	Kessler scale-10	NA	NA	13.36	9
Parcesepe AM et al. [42]	1175	Psychological distress	CS	Oromia	Kessler scale-10	NA	NA	29.5	9
Moges NA et al. [33]	689	Psychological distress	CS	Amhara	Kessler scale-10	NA	NA	58.63	9

### The quality of studies included in the review

Based on the Newcastle–Ottawa Scale, 8 studies were of high methodologic quality, whereas three studies were of moderate methodologic quality. The agreed levels between the authors regarding the quality of the studies included the meta-analysis ranged from moderate to almost perfect levels (Supplementary file 1).

### Meta-analysis

**Fig. 2:**
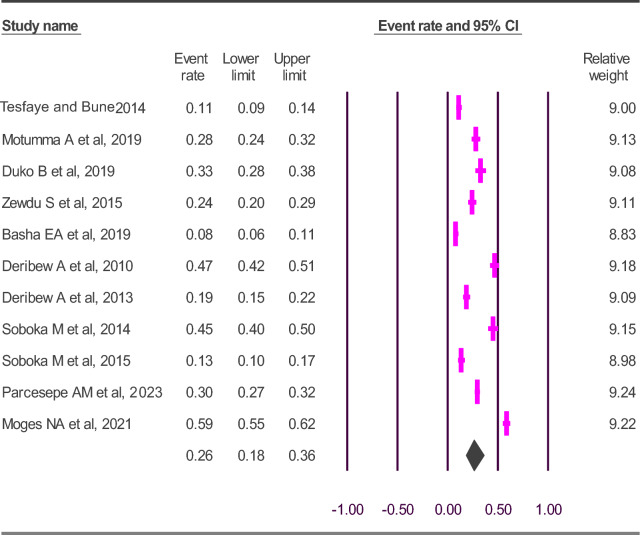
The prevalence of CMD among PLWHA in Ethiopia: a meta-analysis

The overall pooled prevalence estimate of CMD among PLWHA in Ethiopia was 26.1% (95% CI 18.1–36.0), with significant  observed heterogeneity (*I*^2^ = 97.436%; Q = 234.037, df = 6, p < 0.001) (Fig. 2).

### Sensitivity analysis

We performed a leave-one-out sensitivity analysis to identify the possible source of heterogeneity in the pooled meta-analysis of the prevalence of the CMD among PLWHA in Ethiopia. This sensitivity analysis involves performing a meta-analysis on each subset of the studies obtained by leaving out exactly one study at a time. This approach helps to determine how the removal of each study affects the overall estimate of the remaining studies. The pooled prevalence of CMD ranged between 23.5% (16.5–31.7%) and 28.9% (20.5–39.0), suggesting the pooled estimated prevalence of the CMD among PLWHA was not significantly affected by the removal of any single study (Table 2).

**Table 2:** Sensitivity analysis of CMD among PLWHA in Ethiopia for each study being removed at a time: prevalence and 95% confidence

Study excluded	Prevalence %	95% CI
Tesfaye and Bune [17]	28.2	19.8–38.3
Motumma A et al. [28]	25.9	17.3–36.8
Duko B et al. [26]	25.5	17.0–36.2
Zewdu S et al. [25]	26.3	17.7–37.1
Basha EA et al. [24]	28.9	20.5–39.0
Deribew A et al. [27]	24.3	16.3–34.7
Deribew A et al. [29]	26.9	18.4–37.6
Soboka M et al. [30]	24.5	16.4–34.9
Soboka M et al. [31]	27.7	19.2–38.1
Parcesepe AM et al. [42]	25.7	16.6–37.6
Moges NA et al. [33]	23.5	16.5–31.7

Key. The analysis is based on random effect mode

### Meta-regression

In this systematic review and meta-analysis, we first employed univariate regression analysis to guide the selection of the associated factors to include in the final meta-regression model. In the final meta-regression model, we quantified the impacts of the screening tools used to measure the CMD the gender of the study participants (male or female), and the region where the study was originated. The overall proportion of variance explained by the tools used to assess CMD, the gender of the study participants, and the study region was analyzed in the final model. The overall proportion of variance explained by the gender of the study participants and the region the study was conducted were 34% (*R*^2^ = 34%; *P*-value = 0.02) and 6% (*R*^2^ = 6%; *P*-value = 0.34) respectively. Except for the gender of the study participants, neither the tools used to assess the CMD nor the region where the studies were originated were statistically significant determinants for the observed variation in the prevalence of the CMD among studies included in this  review.

### Statistical analysis of factors associated with CMD 

In our statistical analysis of factors associated with CMD among PLWHA  in Ethiopia, we identified  two determinants that were commonly adjusted for in the studies included in this systematic review and meta-analysis. The pooled adjusted odds ratios (AOR) of perceived HIV-related stigma and perceived poor social support on CMD were 2.91, 95% CI (1.35–6.29) and 5.56, 95% CI (1.89–16.39)), respectively (Table 3).

**Table 3:** Factors associated with CMD  among PLWHA that were commonly adjusted in the included studies

Study name	Sample size	Type of outcome	Perceived HIV stigma (AOR, 95% CI)	Poor social support (AOR, 95% CI)
Tesfaye and Bune [17]	500	Psychological distress	NA	10.17 (2.85–36.29)
Motumma A et al. [28]	420	Common mental disorder	NA	NA
Duko B et al. [26]	294	Common mental disorder	1.97 (1.63–2.89)	2.44 (1.33–4.51)
Zewdu S et al. [25]	417	Common mental disorder	7.7 (2.53, 18.8)	NA
Basha EA et al. [24]	422	Psychological distress	2.41 (1.11–5.22)	NA
Deribew A et al. [27]	465	Common mental disorder	NA	10.0 (2.8–35.1)
Deribew A et al. [29]	465	Common mental disorder	NA	NA
Soboka M et al. [30]	389	Common mental disorder	NA	NA
Soboka M et al. [31]	389	Common mental disorder	NA	NA
Parcesepe AM et al. [42]	1175	Psychological distress	NA	NA
Moges NA et al. [33]	689	Psychological distress	1.09 (1.04, 1.15)	NA

### Publication bias

**Fig. 3:**
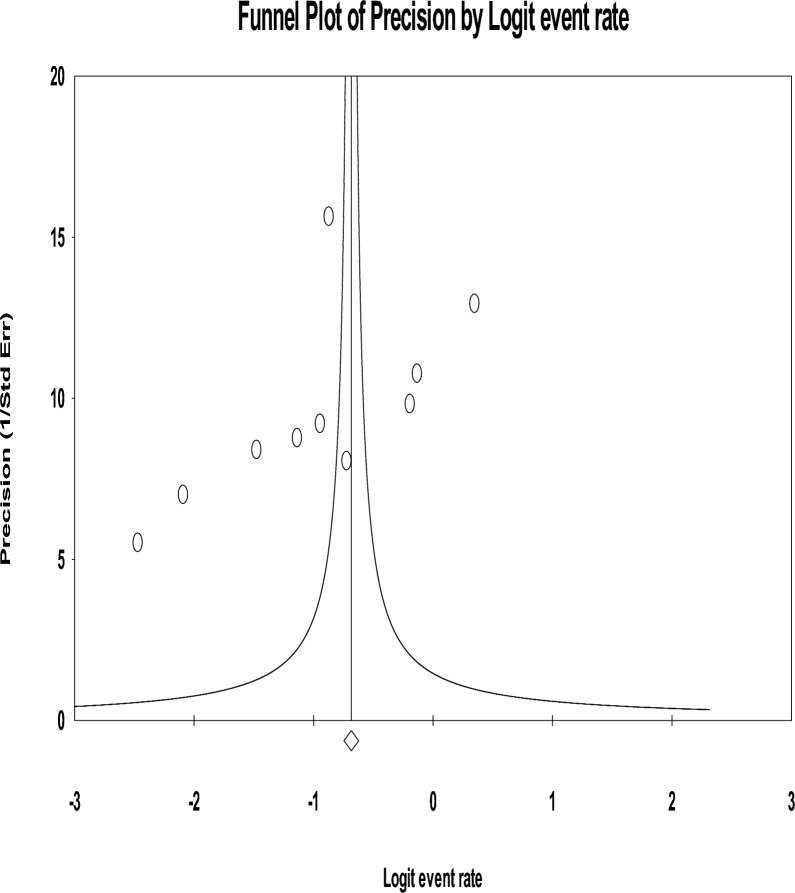
Funnel plot shows no evidence of publication bias among studies included in the meta-analysis

The Egger’s regression test, as well as visual inspection of the funnel plot, showed no evidence of publication bias ((B = − 28.39, SE = 6.45, P = 0.09) (Fig. 3).

## Discussion

In this systematic review and meta-analysis, we examined  the pooled prevalence of CMD among PLWHA in Ethiopia. The final meta-analysis included eleven studies, and the pooled prevalence of CMD among PLWHA was 26.1%. This implies that CMD is a major public health problem among PLWHA in Ethiopia. The pooled prevalence of CMD in this review was in line with a study conducted in South Africa, which reported a prevalence rate of 23.9% (8). A cross-sectional study conducted in southwest regional hospitals of Cameroon among PLWHA on HAART also reported a similar prevalence rate [43]. Furthermore, a study conducted in Ethiopia to assess the prevalence of CMD  among patients with glaucoma reported a similar prevalence rate of (23.2%) [44]. Nevertheless, the pooled estimated prevalence of CMD among PLWHA was lower than the findings of a study  from a Nigerian teaching hospital, which showed a prevalence rate of ( 47.9%) [9]. Further, a study on psychological distress among Ugandan female adolescents living with HIV reported a much higher CMD prevalence rate of 53% [45]. A cross-sectional survey conducted in Zimbabwe at a large primary health care facility found a CMD prevalence rate of 68.5% using locally to identify factors associated with CMD  using locally validated screening tools, namely the Shona Symptom Questionnaire (SSQ-14) [7]. In contrast, the pooled estimated prevalence of CMD was higher than the findings from studies conducted in East Zimbabwe among individuals living with HIV [46] and in the general community of Kenya [45]. The variation in the prevalence of CMD might be attributed to the difference in geographical, cultural, and socio-economy status of the study areas as well as the difference in the study populations. For example, a study conducted in Uganda only included female adolescents aged 12–19 living with HIV [45].

The prevalence of CMD in the current meta-analysis was significantly higher in females (39.5%) compared to males (26.9%). This finding aligns with prior study that has shown a higher prevalence of CMD among females than males [45]. For example, a study assessing the  prevalence of psychological distress among individuals in HIV Care Service Utilization in East Zimbabwe found a prevalence of 4.5% among men and 12.9% among women [46]. This variation may be attributed to specific forms of depression-related illness experienced by women, such as  premenstrual dysphoric disorder, postpartum depression, and postmenopausal mental illness, which are linked with changes in ovarian hormones that may have contributed to the observed difference in the prevalence of CMD among PLWHA  [47, 48].

People living with HIV/AIDS who reported HIV -related perceived stigma were approximately three times more likely to have CMD compared to their counterparts. Of the eleven studies included in the review, four reported statistically significant associations between perceived stigma [26–29]. Individuals who experience stigma may develop a poor self-image and become  socially isolated, which can increase their risk of developing CMD [49]. Furthermore, perceived stigma may lead to internalized prejudice and negative self-perceptions, resulting in decreased self-esteem and further contributing to the progression of CMD PLWHA  [50]. These negative self-perceptions and social isolation can limit social interactions and affect occupational functioning thereby increasing the risk of CMD. 

Furthermore, PLWHA  who reported poor social support were approximately 6 times more likely to have CMD compared to those with better social support.  This finding was supported by three of the studies included in the review [17, 27, 29]. Poor social support can trigger feelings of social isolation and negatively impact both physical and mental well-being [26–28]. This is consistent with the social causation model, which suggests that  a lack of social support increases the likelihood of CMD, such as depression  [51, 52] Conversely, good social support may alleviate CMD by improving self-esteem and reducing negative thoughts  [53, 54].

## Conclusion

This review found that the pooled prevalence of CMD among PLWHA was considerably high. Our findings suggest that PLWHA who receive poor social support and those experiencing HIV-related perceived stigma are at a greater risk of developing CMD. Therefore, it is advisable that all PLWHA attending ART clinics be routinely screened for CMD, social support,  and HIV-related perceived stigma. In addition to this, health professionals, more specifically, clinical psychologists and mental health professionals should provide regular counselling to enhance stress-coping mechanisms and improve the mental well-being of PLWHA attending ART clinics. Finally, we recommend that researchers consider conducting a large -scale longitudinal studies to further explore the burden and risk factors of CMD among PLWHA.

### Limitations of the review

The following are the limitations of our systematic review and meta-analysis that should be  considered when interpreting our findings. First, only eleven studies published in the past ten years met the inclusion criteria. Second, due to variations in diagnostic approaches, the tools used to screen CMD may be prone to measurement bias. However, we have addressed this issue of heterogeneity during our analysis, which provides more reliable estimate of CMD and its associated factors among PLWHA in Ethiopia.

### Supplementary Information

Supplementary Material 1. Summary of the quality and agreed level of bias and level of agreement on the methodological qualities of included studies in a meta-analysis.

## Data Availability

All data generated or analyzed during this study are included in this article.

## Abbreviations

AIDS: Acquired immune deficiency syndrome
AJOL: African journal of online
AOR: Adjusted odds ratio
ART: Anti-retroviral therapy
CI: Confidence interval
CMD: Common mental disorder
HADS: Hospital anxiety and depression scale
HIV: Human immune virus
K-10: Kessler scale
NOS: Newcastle–Ottawa Scale
PLWHA: People living with HIV/AIDS
PRISMA: Preferred reporting items for systematic reviews and meta-analyses
PTSD: Post-traumatic stress disordersrq-20: self-reporting questionnaire item 20
SSQ-14: Shona symptom questionnaire item 14

## References

[1] Lopez AD, Mathers CD, Ezzati M, Jamison DT, Murray CJ (2006). Global burden of disease and risk factors.

[2] Steel Z, Marnane C, Iranpour C, Chey T, Jackson JW, Patel V (2014). The global prevalence of common mental disorders: a systematic review and meta-analysis 1980–2013. Int J Epidemiol.

[3] Prince M, Patel V, Saxena S, Maj M, Maselko J, Phillips MR (2007). No health without mental health. Lancet.

[4] Mayston R, Kinyanda E, Chishinga N, Prince M, Patel V (2012). Mental disorder and the outcome of HIV/AIDS in low-income and middle-income countries: a systematic review. AIDS.

[5] Sherr L, Clucas C, Harding R, Sibley E, Catalan J (2011). HIV and depression–a systematic review of interventions. Psychol Health Med.

[6] Olagunju AT, Adeyemi JD, Ogbolu RE, Campbell EA (2012). A study on epidemiological profile of anxiety disorders among people living with HIV/AIDS in a sub-Saharan Africa HIV clinic. AIDS Behav.

[7] Chibanda D, Cowan F, Gibson L, Weiss HA, Lund C (2016). Prevalence and correlates of probable common mental disorders in a population with high prevalence of HIV in Zimbabwe. BMC Psychiatry.

[8] Mthembu JC, Mabaso M, Khan G, Simbayi LC (2017). Prevalence of psychological distress and its association with socio-demographic and HIV-risk factors in South Africa: findings of the 2012 HIV prevalence, incidence and behaviour survey. SSM - Popul Health.

[9] Igwe MN, Ndukuba AC, Olose EO, Obayi NO, Ajayi NA, Odo A, Njoku PO, Amadi KU, Ezeme MS, Aguocha C (2016). Psychological distress and quality of life of people living with HIV/AIDS in a Nigerian teaching hospital. Ment Health Relig Cult.

[10] Patel V, Araya R, De Lima M, Ludermir A, Todd C (1999). Women, poverty and common mental disorders in four restructuring societies. Soc Sci Med.

[11] Freeman M, Nkomo N, Kafaar Z, Kelly K (2007). Factors associated with prevalence of mental disorder in people living with HIV/AIDS in South Africa. AIDS Care.

[12] Kinyanda E, Hoskins S, Nakku J, Nawaz S, Patel V (2011). Prevalence and risk factors of major depressive disorder in HIV/AIDS as seen in semi-urban Entebbe district, Uganda. BMC Psychiatry.

[13] Olley B, Seedat S, Nei D, Stein D (2004). Predictors of major depression in recently diagnosed patients with HIV/AIDS in South Africa. AIDS Patient Care STDS.

[14] Adewuya AO, Afolabi MO, Ola BA, Ogundele OA, Ajibare AO, Oladipo BF (2009). Post-traumatic stress disorder (PTSD) after stigma related events in HIV infected individuals in Nigeria. Soc Psychiatry Psychiatr Epidemiol.

[15] Akena D, Musisi S, Joska J, Stein DJ (2012). The association between aids related stigma and major depressive disorder among HIV-positive individuals in Uganda. PLoS ONE.

[16] Rao D, Kekwaletswe T, Hosek S, Martinez J, Rodriguez F (2007). Stigma and social barriers to medication adherence with urban youth living with HIV. AIDS Care.

[17] Tesfaye SH, Bune GT (2014). Generalized psychological distress among HIV-infected patients enrolled in antiretroviral treatment in Dilla University Hospital, Gedeo zone, Ethiopia. Glob Health Action.

[18] Halman M, Chan Carusone S, Stranks S, Schaefer-McDaniel N, Stewart A (2014). Complex care needs of patients with late-stage HIV disease: a retrospective study. AIDS Care.

[19] Antelman G, Kaaya S, Wei R, Mbwambo J, Msamanga GI, Fawzi WW (2007). Depressive symptoms increase risk of HIV disease progression and mortality among women in Tanzania. J Acquir Immune Defic Syndr.

[20] Paterson DL, Swindells S, Mohr J, Brester M, Vergis EN, Squier C (2000). Adherence to protease inhibitor therapy and outcomes in patients with HIV infection. Ann Intern Med.

[21] Leserman J (2008). Role of depression, stress, and trauma in HIV disease progression. Psychosom Med.

[22] Ironson G, O’Cleirigh C, Fletcher MA, Laurenceau JP, Balbin E, Klimas N (2005). Psychosocial factors predict CD4 and viral load change in men and women with human immunodeficiency virus in the era of highly active antiretroviral treatment. Psychosom Med.

[23] Ickovics JR, Hamburger ME, Vlahov D, Schoenbaum EE, Schuman P, Boland RJ (2001). Mortality, CD4 cell count decline, and depressive symptoms among HIV-seropositive women: longitudinal analysis from the HIV epidemiology research study. JAMA.

[24] Basha EA, Derseh BT, Haile YGE, Tafere G (2019). Factors affecting psychological distress among people living with HIV/AIDS at selected hospitals of north Shewa zone, Amhara region, Ethiopia. AIDS Res Treat.

[25] Zewdu S, Abebe N (2015). Common mental disorder among HIV infected individuals at comprehensive HIV care and treatment clinic of Debre Markos referral hospital, Ethiopia. J AIDS Clin Res.

[26] Duko B, Toma A, Abraham Y (2019). Prevalence and correlates of common mental disorder among HIV patients attending antiretroviral therapy clinics in Hawassa City, Ethiopia. Ann Gen Psychiatry.

[27] Deribew A, Tesfaye M, Hailmichael Y (2010). Common mental disorders in TB/HIV co-infected patients in Ethiopia. BMC Infect Dis.

[28] Motumma A, Negesa L, Hunduma G, Abdeta T (2019). Prevalence and associated factors of common mental disorders among adult patients attending HIV follow up service in Harar town, Eastern Ethiopia: a cross-sectional study. BMC Psychol.

[29] Deribew A, Deribe K, Reda AA, Tesfaye M, Hailmichael Y, Maja T (2013). Do common mental disorders decline over time in TB/HIV co-infected and HIV patients without TB who are on antiretroviral treatment?. BMC Psychiatry.

[30] Soboka M, Tesfaye M, Feyissa GT (2014). Alcohol use disorders and associated factors among people living with HIV who are attending services in south west Ethiopia. BMC Res Notes.

[31] Soboka M, Tesfaye M, Feyissa GT (2015). Khat use in people living with HIV: a facility-based cross-sectional survey from South West Ethiopia. BMC Psychiatry.

[32] Gadisa T, Kulkarni SG, Hoffman S, Melaku Z, Elul B, Nash D (2018). Psychological distress, health and treatment-related factors among individuals initiating ART in Oromia, Ethiopia. AIDS Care.

[33] Moges NA, Adesina OA, Okunlola MA, Berhane Y, Akinyemi JO (2021). Psychological distress and its correlates among newly diagnosed people living with HIV in Northwest Ethiopia: ordinal logistic regression analyses. Infect Dis: Res Treat.

[34] Moher D, Shamseer L, Clarke M, Ghersi D, Liberati A, Petticrew M (2015). Preferred reporting items for systematic review and meta-analysis protocols (PRISMA-P) 2015 statement. Syst Rev.

[35] Stang A (2010). Critical evaluation of the Newcastle-Ottawa scale for the assessment of the quality of nonrandomized studies in meta-analyses. Eur J Epidemiol.

[36] Duko B, Wolka S, Seyoum M (2021). Prevalence of depression among women with obstetric fistula in low-income African countries: a systematic review and meta-analysis. Arch Womens Ment Health.

[37] Duko B, Wolde D, Alemayehu Y (2020). The epidemiology of postnatal depression in Ethiopia: a systematic review and meta-analysis. Reprod Health.

[38] Landis J, Koch G (1977). The measurement of observer agreement for categorical data. Biometrics.

[39] Borenstein M, Hedges LV, Higgins J, Rothstein HR (2010). A basic introduction to fixed-effect and random-effects models for meta-analysis. Res Synth Methods.

[40] Higgins JP, Thompson SG, Deeks JJ, Altman DG (2003). Measuring inconsistency in meta-analyses. BMJ: Br Med J.

[41] Sterne JAC, Gavaghan D, Egger M (2000). Publication and related bias inmeta-analysis: power of statistical tests and prevalence in the literature. J Clin Epidemiol.

[42] Parcesepe AM, Stockton M, Remch M, Wester CW, Bernard C, Ross J, et al. Availability of screening and treatment for common mental disorders in HIV clinic settings: data from the global International epidemiology Databases to Evaluate AIDS (IeDEA) Consortium, 2016–2017 and 2020. J Int AIDS Soc. 2023;26:e26147. 10.1002/jia2.26147. 10.1002/jia2.26147 PMC10399924 37535703

[43] Ngum PA (2017). Depression among HIV/AIDS patients on highly active antiretroviral therapy in the southwest regional hospitals of Cameroon: a cross-sectional study. Neurol Ther.

[44] Bedasso K, Bedaso A, Feyera F, Gebeyehu A, Yohannis Z (2016). Prevalence of common mental disorders and associated factors among people with glaucoma attending outpatient clinic at Menelik II Referral Hospital, Addis Ababa, Ethiopia. PLoS ONE.

[45] Mutumba M, Bauermeister JA, Harper GW, Musiime V, Lepkowski J, Resnicow K (2017). Psychological distress among Ugandan adolescents living with HIV: examining stressors and the buffering role of general and religious coping strategies. Glob Public Health.

[46] Tlhajoane M, Eaton JW, Takaruza A, Rhead R, Maswera R, Schur N, Gregson S (2018). Prevalence and associations of psychological distress, HIV infection and HIV care service utilization in East Zimbabwe. AIDS Behav.

[47] Jenkins R, Njenga F, Okonji M, Kigamwa P, Baraza M, Ayuyo J (2012). Prevalence of common mental disorders in a rural district of Kenya, and socio-demographic risk factors. Int J Environ Res Public Health.

[48] Albert PR (2015). Why is depression more prevalent in women?. J Psychiatry Neurosci: JPN.

[49] Nüesch R, Gayet-Ageron A, Chetchotisakd P, Prasithsirikul W, Kiertiburanakul S, Munsakul W (2009). The impact of combination antiretroviral therapy and its interruption on anxiety, stress, depression and quality of life in Thai patients. Open AIDS J.

[50] Tucker JR, Hammer JH, Vogel DL, Bitman RL, Wade NG, Maier EJ (2013). Disentangling self-stigma: are mental illness and help-seeking self-stigmas different?. J Couns Psychol.

[51] Kaniasty K, Norris FH (2008). Longitudinal linkages between perceived social support and posttraumatic stress symptoms: sequential roles of social causation and social selection. J Trauma Stress.

[52] Zhen R, Quan L, Zhou X (2018). How does social support relieve depression among survivors after massive flood? The contribution of feelings of safety, self-disclosure, and posttraumatic cognition. J Affect Disord.

[53] Abas M, Broadhead JC, Mbape P, Khumalo-Sakatukwa G (1994). Defeating depression in the developing world: a Zimbabwean model: one country's response to the challenge. Br J Psychiatry.

[54] Zang Y, Gallagher T, McLean CP, Tannahill HS, Yarvis JS, Foa EB (2017). The impact of social support, unit cohesion, and trait resilience on PTSD in treatment-seeking military personnel with PTSD: the role of posttraumatic cognitions. J Psychiat Res.

