# Supplementary material for: Prevalence of common mental disorder and its association with perceived stigma and social support among people living with HIV/AIDS in Ethiopia: a systematic review and meta-analysis
Source: Int J Ment Health Syst. 2024 Jul 8;18:25. doi: 10.1186/s13033-024-00641-x (PMC11232219; doi:10.1186/s13033-024-00641-x)
Supplement: Supplementary file 1 — Supplementary Material 1. Summary of the quality and agreed level of bias and level of agreement on the methodological qualities of included studies in a meta-analysis. [file 13033_2024_641_MOESM1_ESM.docx]

**Supplemenraty file 1;** Summary of the quality and agreed level of bias and level of agreement on the methodological qualities of included studies in a meta-analysis

| Study | Overall agreement and precision | | | Nos quality (score on 0 to 9 scale) |
| --- | --- | --- | --- | --- |
|  | Percentage of agreement | Kappa value | Level of agreement |  |
| Solomon H et al, 2014 | 100 | 1 | Almost perfect | 8 |
| Motumma A et al, 2019 | 75 | 0.60 | Moderate | 8 |
| Duko B et al, 2019 | 100 | 1 | Almost perfect | 9 |
| Zewdu S et al, 2015 | 75 | 0.60 | Moderate | 7 |
| Basha EA et al, 2019 | 75 | 0.60 | Moderate | 7 |
| Deribew A et al, 2010 | 100 | 1 | Almost perfect | 9 |
| Deribew A et al, 2013 | 100 | 1 | Almost perfect | 9 |
| Soboka M et al, 2014 | 75 | 0.60 | Moderate | 7 |
| Soboka M et al, 2015 | 100 | 1 | Almost perfect | 9 |
| Parcesepe AM et al, 2018 | 100 | 1 | Almost perfect | 9 |
| Moges NA et al, 2021 | 100 | 1 | Almost perfect | 9 |
